# An Integrative Drug‐Induced Transcriptomic Analysis Identifies Novel MYC Antagonists and Potential Synergistic Drug Combinations

**DOI:** 10.1002/mc.70044

**Published:** 2025-09-30

**Authors:** Anthony Aceto, Yue Wang, Da Yang

**Affiliations:** ^1^ Center for Pharmacogenetics, Department of Pharmaceutical Sciences University of Pittsburgh Pittsburgh USA; ^2^ UPMC Hillman Cancer Center University of Pittsburgh Pittsburgh USA; ^3^ Department of Computational and System Biology University of Pittsburgh Pittsburgh USA

**Keywords:** drug combinations, in‐silico screening, MYC, post‐perturbation profiling

## Abstract

MYC is among the most frequently dysregulated oncogenes in human cancer, yet its direct targeting remains a significant challenge. Here, we present an in‐silico integrative screening approach to identify compounds and combinations that can block MYC's oncogenic function by specifically disrupting its transcriptional regulatory function. Using a doxycycline (DOX)‐inducible model, we established a MYC loss‐of‐function (LOF) gene signature that specifically captures the molecular consequences corresponding to the loss of MYC's ability in transcriptional regulation. By integrating large‐scale post‐perturbation transcriptomic profiling from the CMAP database, we screened over 8300 drug‐induced profiles and identified 70 recurrent compounds that are predicted to antagonize MYC's transcriptional programs. To further enhance their therapeutic potential, we also developed an orthogonality analysis to pinpoint synergistic drug combinations that suppress MYC activity more effectively than single agents. Our scalable framework enables a rational and systematic identification of compounds with potential to antagonize MYC's oncogenic function by disrupting its transcriptional regulatory ability without necessarily decreasing its abundance. Our approach provides new insights on utilizing existing anticancer drugs to indirectly target MYC in MYC‐driven cancer.

## 1 Introduction

The MYC oncogene is one of the most frequently dysregulated drivers in human cancer, promoting uncontrolled proliferation, altered metabolism, immune evasion, and other hallmark features of malignancy [1, 2]. As a transcription factor, MYC regulates a broad network of genes by heterodimerizing with MAX and binding E‐box DNA elements, enabling it to orchestrate oncogenic transcriptional programs [3, 5]. Many tumors exhibit “MYC addiction”, wherein sustained MYC activity is essential for tumor maintenance and progression [1, 2, 4]. Since MYC dysregulation is observed in approximately 70% of all cancers and plays a causal role in cancer progression, it is a promising target for anticancer therapy [6, 7, 8].

Despite its appeal as a therapeutic target, direct inhibition of MYC remains clinically elusive due to a lack of a definitive active site. Small molecules that disrupt MYC‐MAX dimerization, such as 10058‐F4, show efficacy in vitro but are limited in vivo by poor pharmacokinetic and pharmacodynamic properties [9, 10]. As a result, recent efforts have focused on indirect strategies that suppress MYC‐driven transcriptional activity through chromatin regulators. Bromodomain and extra‐terminal motif (BET) inhibitors such as JQ1 impair transcriptional initiation at MYC‐bound enhancers, while histone deacetylase inhibitors (HDACi) such as vorinostat reduce MYC expression epigenetically [10, 11]. Given the pleiotropic effects of MYC, monotherapies may be insufficient to fully suppress its oncogenic functions. Combination therapies targeting complementary pathways could enhance efficacy, reduce resistance, and mitigate toxicity by enabling lower effective doses of individual agents [12, 13, 14].

Our recent study established a DOX‐inducible MYC‐mutant‐MYC‐KO system in cancer cell line, which enables the accurate profiling of transcriptional consequences of MYC's loss‐of‐function (LOF) on its RNA and DNA binding ability [15]. Using this system, we derived a MYC LOF signature that specifically characterizes the decoupling of MYC's function from its abundance through blocking its transcriptional regulatory activity. Using this signature, we leveraged the large‐scale post‐perturbation transcriptomic profiling by LINCS L1000 Connectivity Map (CMAP) [16] to systematically screen for compounds that can disrupt MYC's transcriptional regulatory function. This analysis identified 70 highly confident compounds that potentially antagonize MYC's transcriptional programs [12]. Building on these findings, we further evaluated the top single‐agent hits in pairwise combinations to uncover synergistic potential to maximize therapeutic impact. By integrating gene‐level and pathway‐centric analyses, this study implements a rational and systematic approach to identify compounds with potential to disrupt MYC's transcriptional regulation activity without necessarily changing MYC's expression, which provides new insights on utilizing existing anticancer drugs to target MYC in MYC‐driven cancers.

## 2 Methods

### 2.1 Data Acquisition

Chemical perturbation transcriptomic data were obtained from the LINCS L1000 Level 5 data set via https://clue.io/. These data represent replicate‐averaged, normalized Z‐scores of gene expression changes relative to negative controls. Only signatures with Transcriptional Activity Scores (TAS) ≥ 0.4 were retained to ensure sufficient transcriptional response strength and reproducibility [17]. Each signature includes expression measurements for 978 landmark genes and ~12,000 computationally inferred genes.

Additional cell line data were retrieved from three publicly available sources. Gene expression profiles and metadata for 1019 cancer cell lines were obtained from the Cancer Cell Line Encyclopedia (CCLE) [18]. Half‐maximal inhibitory concentration (IC₅₀) drug sensitivity values were sourced from the Genomics of Drug Sensitivity in Cancer database (GDSC2) [19]. Gene dependency scores were acquired from the DepMap portal [20], which provides genome‐wide CRISPR–Cas9 knockout screen data. These scores estimate the probability that a gene is essential for cellular proliferation or survival in a given cell line.

### 2.2 RNA Sequencing and Differential Expression Analysis

A MYC loss‐of‐function (LOF) mutant, designated KO7A, was used in U2OS cells with doxycycline (DOX)‐inducible expression, as previously described [15]. DOX+ and DOX− conditions respectively correspond to the presence and absence of MYC mutant induction. The KO7A construct has been shown to impair both RNA and DNA binding functions of MYC, effectively decoupling its transcriptional activity. Next, RNA sequencing was performed on U2OS DOX‐inducible cell lines, with raw FASTQ files processed using the nf‐core RNA‐seq pipeline (v3.18.10). Alignment was conducted using STAR RSEM (v2.7.11b) against the reference genome (GRCh38) annotated with Gencode v46, and quality control was assessed via MultiQC (v1.25.1). Resulting BAM files were then analyzed with CuffDiff2 for pairwise differential expression analysis of MYC mutants. Genes were considered differentially expressed if they met the thresholds of |log2(fold change)| > 1 and *p*‐value ≤ 0.05.

### 2.3 Signature Construction

For this analysis, signatures were constructed from differential expression profiles reflecting the transcriptomic effects of perturbation. Because CMAP L1000 signatures are expressed as Z‐scores and MYC LOF signatures as log2(fold change), a binarization procedure was applied to standardize gene‐level regulation across platforms. If absolute log2(fold change) ≥ 1 or absolute *Z*‐score ≥ 1, the gene was assigned to 1 (upregulated) or −1 (downregulated), respectively. Genes below these thresholds were assigned 0 (nonsignificant).

### 2.4 Normalized Concordance Score

**Figure 1:**
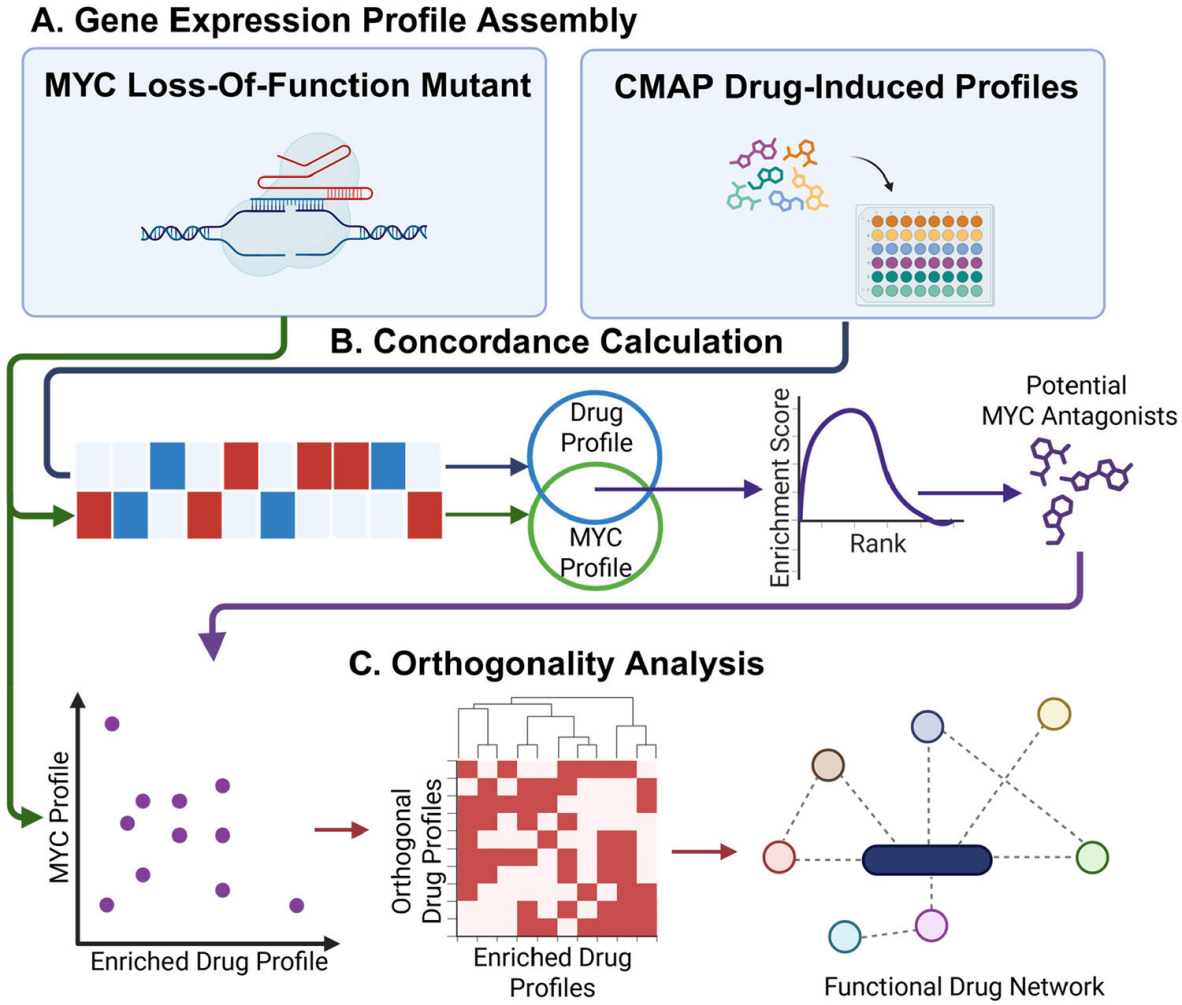
The computational framework to characterize MYC antagonists and synergistic partners. (A) Identification of MYC LOF signature in U2OS and drug signatures across 10 cell lines with 10uM and 24 h perturbation time points. (B) Concordance is calculated between CMAP compounds and the MYC LOF target profile to produce single agents with the highest concordance to the MYC LOF target profile. (C) Orthogonality analysis to construct a drug combination network that models functionally similar synergistic effects against MYC activity.

Similar to the approach outlined by Stathias et al [12], our analysis compares drug‐induced expression signatures to a MYC LOF profile (Figure 1A) by quantifying both the overlap of DEGs and the concordance of their expression direction (Figure 1B).

Let G be the set of all genes, and let profiles $A=\left\{a_{1},a_{2},\ldots ,a_{n}\right\}$ and $B=\left\{b_{1},b_{2},\ldots ,b_{n}\right\}$ represent binarized gene expression values for a reference and drug‐induced signature, respectively, where each $a_{i},b_{i}\in \{-1,0,1\}$. Here, ±denotes significant up/downregulation, and 0 denotes non‐significance.

The set of overlapping DEGs is defined as $O=\left\{g\in G\right|a_{g}\neq 0\wedge b_{g}\neq 0\}$. Within O, the concordant subset C includes genes for which $a_{g}=b_{g}$, and the disconcordant subset D includes those where $a_{g}\neq b_{g}$.

The Normalized Concordance Ratio (NCR) is defined as:

$$\mathrm{NCR}=\frac{\left|C\right|-|D|}{|A|}$$

Where A is the set of DEGs in the reference profile A. The NCR ranges from ‐1 (fully discordant) to 1 (perfectly concordant).

To assess significance, we performed 1,000 permutations per profile by randomly shuffling gene labels within each drug‐induced signature, preserving the number of up‐ and downregulated genes [21]. The NCR was recalculated for each permuted profile to generate a null distribution. *P*‐values were derived from this distribution and corrected for multiple comparisons using the Benjamini‐Hochberg FDR method.

### 2.5 Drug‐Level Enrichment Analysis

We compared each drug's transcriptional signatures to a reference profile using a GSEA‐style approach [22] adapted for compound‐level analysis. For drugs with multiple replicate signatures, we ranked all signatures by their similarity to the reference. The enrichment score (ES) was calculated by moving down this ranked list, increasing the running sum when a replicate signature for the drug was found and decreasing it otherwise. The ES was the maximum deviation from zero, indicating whether the drug's signatures clustered toward the top or bottom of the list. Statistical significance was assessed by 1,000 permutations, with scores normalized for replicate count. P‐values were corrected for multiple testing using the Benjamini–Hochberg method [22].

### 2.6 Orthogonality Score

Following the framework of Stathias et al [12], we defined an Orthogonality Score (OS) to identify compounds that exhibit strong concordance with the reference target profile while acting through mechanisms distinct from a given drug profile (Figure 1C). The OS integrates concordance metrics as:

$$\mathrm{OS}=\sqrt{{{(\mathrm{NCR}}_{\mathrm{Target}})}^{2}+{\left(1-{\mathrm{NCR}}_{\mathrm{Drug}}\right)}^{2}}$$

where ${\mathrm{NCR}}_{\mathrm{Target}}$ is the normalized concordance ratio between the compound and the reference target profile, and ${\mathrm{NCR}}_{\mathrm{Drug}}$ is the normalized concordance ratio between the compound and the reference drug profile. A higher OS indicates that the compound shares similar transcriptomic effects with the target profile but differs substantially from the reference drug, suggesting potential orthogonal mechanism of action.

### 2.7 Calculating Aggregate Signatures

The L1000 level 5 data set was filtered for only 24 h 10uM treatment samples. Gene expression profiles were aggregated for samples using the same molecule in MCF7. Aggregation was performed by first binarizing each replicate drug‐induced profile then calculating the mode individually for each gene.

## 3 Results

### 3.1 MYC DNA and RNA Binding LOF Impedes MYC Activity

**Figure 2:**
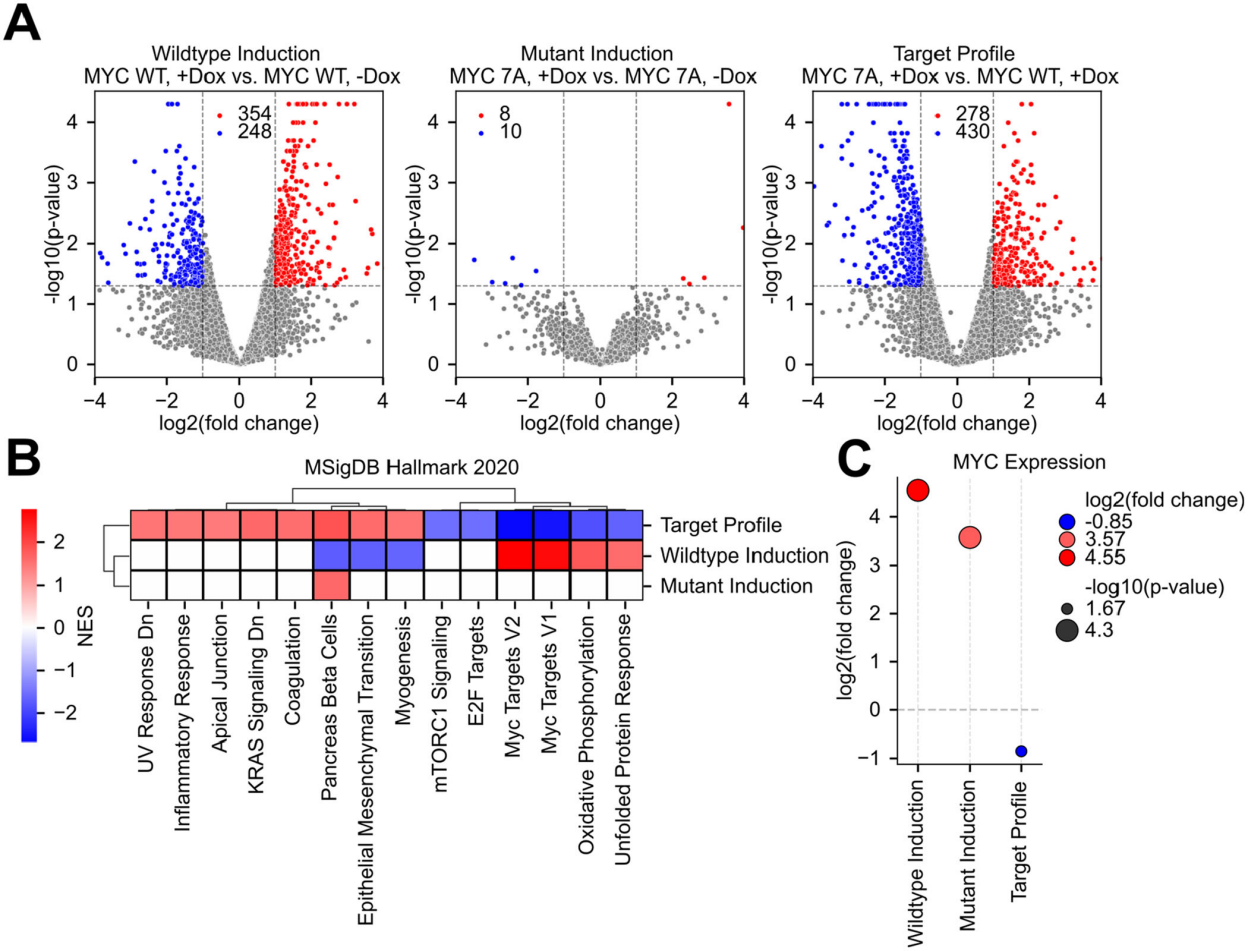
MYC DOX induction and differential expression profiles. (A) Differential expression was defined as fold change > 2 with *p* ≤ 0.05. A Wildtype MYC (KOWT) and MYC loss‐of‐function mutant (KO7A) with and without doxycycline (DOX) induction, alongside a curated target profile representing a desirable drug phenotype that decouples MYC expression from its transcriptional activity. (B) Normalized enrichment scores (NES) from GSEA of MSigDB Hallmark 2020 pathways with FDR ≤ 0.05. (C) Gene‐level MYC expression across all profiles, with point size scaled by −log10(*p*‐value) and color coded by the magnitude of log2(fold change).

We generated a U2OS cell line with an endogenous MYC knockout (MYC‐KO‐U2OS) and introduced a doxycycline (DOX)‐inducible system to overexpress either wildtype MYC (KOWT) or a loss‐of‐function mutant (KO7A) defective in DNA and RNA binding (Figure 2A; see **Methods**). DOX induction of KOWT amplified downstream regulatory effects, whereas KO7A triggered attenuated transcriptional responses, confirming that MYC's activity relies on nucleic acid binding.

These findings highlight the therapeutic potential of strategies that selectively decouple MYC expression from its transcriptional activity, which may lead to the cessation of cancer cell proliferation. We therefore generated a MYC LOF target profile that inversely relates to disease‐associated, MYC wildtype expression patterns, serving as a reference for identifying compounds that mimic MYC's functional decoupling effects (Figure 2A).

To assess the global transcriptional impact of MYC perturbation, we performed gene set enrichment analysis (GSEA) using the MSigDB Hallmark 2020 collection across all three expression profiles. Compared to wildtype MYC induction (KOWT DOX+ *vs.* DOX − , “wildtype induction profile”), 7A‐mutant MYC (KOWT DOX+ *vs.* KO7A DOX + , “target profile”) reverses most of the key pathways of MYC‐related biology while specifically reflecting the molecular consequences of decoupling MYC's transcriptional regulatory function (Figure 2B). Compared to the non‐induced control, induction of the 7A‐mutant MYC (KO7A DOX+ *vs.* DOX − , “mutant induction profile”) showed no statistically significant enrichment in MYC‐related pathways, confirming that the 7A‐mutant MYC, though being highly activated and expressed in cells, has lost most of its transcriptional regulation activity (Figure 2C).

These results suggest that, although DOX induction strongly increases MYC expression, the 7A‐mutant MYC fails to elicit the expected transcriptional regulatory function because of the loss of DNA/RNA‐binding ability. This finding indicates that MYC's oncogenic function can be effectively decoupled from its actual abundance if its transcriptional regulation activity is disrupted. Building on this hypothesis, we sought to identify anticancer drugs that can antagonize MYC's oncogenic function by disrupting its transcriptional regulatory function instead of decreasing its abundance in cells.

### 3.2 Drug‐Level Concordance Enrichment Reveals 70 MYC‐Specific Antagonists

To this end, we sought to screen for drugs that can copy the molecular phenotypes as those in MYC 7A‐mutant versus MYC WT, i.e., the MYC target profile (Figure 2A and B). To evaluate whether a drug can induce similar gene expression changes as in the MYC target profile, we employed a normalized concordance score (see **Methods**). This score integrates both the magnitude and direction of differential gene expression and is normalized to the size of the MYC target profile. Compounds with high positive concordance scores have substantial overlap in DEGs with the same direction as the MYC LOF target profile. These compounds are believed to copy the molecular phenotype of MYC LOF, which is hypothesized to suppress MYC's transcriptional regulation activities and confer anticancer effects in MYC‐driven malignancies.

**Figure 3:**
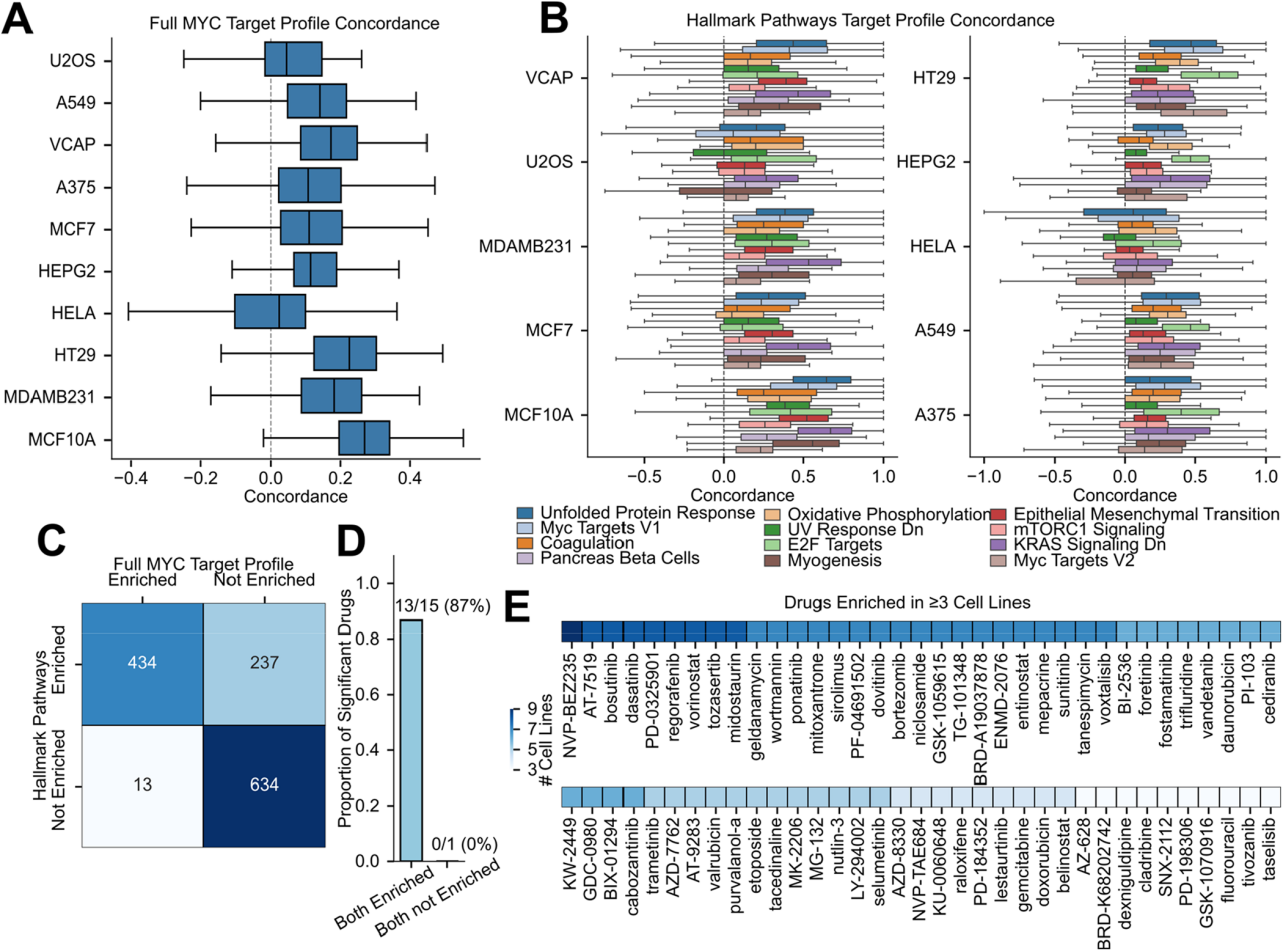
Concordance screening identifies therapeutic candidates with MYC‐dependent sensitivity. (A) Concordance distributions in the full MYC target profile model for the target phenotype. (B) Concordance distributions for 12 significantly enriched pathways in the target profile. (C) Compounds were assessed using a GSEA‐style enrichment analysis; those with NES > 0 and FDR ≤ 0.05 after 1000 permutations in either the full MYC target profile or the hallmark pathway method were classified as enriched, while others were considered not enriched. Results show global frequencies across all cell lines, with Fisher's exact test yielding an odds ratio of 89.31 (*p* = 3.15 × 10⁻¹⁵¹, α ≤ 0.05). (D) For drugs enriched or not enriched in ≥ 6 CMAP cell lines, a Mann–Whitney *U*‐test compared IC₅₀ values between the top and bottom 35% of MYC Target V1 expression. (E) Drugs enriched in ≥ 3 cell lines are shown.

To ensure consistency, we focused our analyses on signatures generated at a dosage of 10 µM and a perturbation time of 24 h (Supporting Figure 1). Additionally, only drug signatures with sufficient transcriptional activity score were included to ensure high‐quality data (see Methods). Cell lines were selected based on a high abundance of unique compounds across multiple tissue types. Initially, we performed signature level screening using all 728 genes which were identified as differentially expressed in the MYC target profile (Figure 3A). The resulting concordance scores displayed a narrower distribution than the theoretical range of (−1, 1), suggesting the complexity of MYC functions that no single drug can fully reverse all of them.

Recognizing that drugs often modulate specific biological pathways rather than the entire transcriptome, we further adopted a pathway‐focused approach to achieve a higher functional resolution and biological interpretability. We selected 12 pathways from the MSigDB Hallmark 2020 collection that showed most significant enrichment in the MYC LOF target profile (Figure 2B). For each drug, concordance scores were recalculated for these pathways, yielding a multidimensional concordance profile that captures pathway‐specific alignment with the MYC LOF phenotype (Figure 3B).

This pathway‐focused analysis increased the dynamic range of concordance scores, bringing values closer to the theoretical limits and enabling stratification of compounds by pathway (Figure 3B). To assess the overall distribution of pathway concordance across cell lines, we quantified the proportion of highly concordant drugs within each pathway relative to the total number of drugs scored for that cell line (Supporting Figure 2A). Pathways downregulated in the MYC LOF profile consistently had higher ratios of high‐concordance drugs across cell lines. Examining the absolute counts of high‐concordance drugs per pathway also revealed that these same downregulated pathways also contain significantly more high‐concordance compounds (*p* = 1.43 × 10^−4^, Student's *t* test; Supporting Figure 2B) To further investigate whether these pathways are relevant to cancer cell survival, we integrated the gene dependency score from DepMap [20] (Supporting Figure 2C). We observed that downregulated pathways uniformly showed high gene dependency, suggesting drugs that suppress these pathways are likely to induce cell death or inhibit aggressive proliferation.

To compare the pathway‐focused and full MYC target profile analyses in identifying significantly concordant compounds, we performed a GSEA‐like enrichment analysis [22] (see Methods) on the concordance score rankings for each cell line. Compounds with normalized enrichment scores (NES) > 0 and false discovery rate (FDR) ≤ 0.05 were classified as enriched. Comparison of enrichment labels between the pathway‐centric and full MYC Target Profile signature methods showed substantial overlap (odds ratio = 89.31, *p* = 3.15 × 10^−151^, Fisher's exact test), with the pathway‐focused analysis identifying an additional 237 enriched compounds (Figure 3C). These results indicate that these two methods show strong global and cell‐line level consistency in identifying enriched compounds.

Next, to prioritize potent candidate MYC antagonists, compounds enriched in at least three CMAP cell lines by both methods were selected (Figure 3E). This pool included known indirect MYC antagonists such as vorinostat (an HDAC inhibitor), rapamycin (an mTOR inhibitor), geldanamycin (an HSP90 inhibitor), and LY‐294002 (PI3K/BET bromodomain dual inhibitor) [23, 24, 25, 26]. Compounds such as NVP‐BEZ235 (Dactolisib) (dual PI3K/mTOR inhibitor) have been previously shown by [13, 27] to potently induce apoptosis in MYC‐driven cell lines and in vivo. Similarly, although AT‐519 is identified as a CDK inhibitor [28], demonstrate that, in a time dependent manner, AT‐519 decreases MYC protein levels after 6 h at 0.5uM via GSK‐3β activation and RNA polymerase II inhibition. PD‐0325901, a novel MEK inhibitor, was also shown to suppress MYC function through inhibiting ERK phosphorylation in vitro and in vivo [29]. These results suggest that our approach can recapitulate previously identified indirect inhibitors of MYC.

To further validate our findings, we integrated the drug responses data from the Cancer Cell Line Encyclopedia and Genomics of Drug Sensitivity in Cancer databases [18, 19]. We hypothesize that if a drug can induce similar molecular phenotype of MYC LOF and thereby suppresses MYC's function, cell lines with high MYC dependency should exhibit higher sensitivity to this drug compared to those with low MYC dependency. In this analysis, we focused on 15 MYC antagonist candidates that show MYC suppression potential in six or more cell lines (Supporting Figure 3A). We stratified cell lines by MYC Target V1 signature enrichment (top and bottom 35%) from the Cancer Cell Line Encyclopedia [18] (Supporting Figure 3B) and compared the drug IC50s between two cell line groups (Figure 3D). Among the 15 MYC antagonist candidates, 87% of them exhibited significantly greater sensitivity in high MYC activity cell lines, providing strong evidence that their therapeutic effect is dependent or partially dependent on MYC function.

### 3.3 Network Analysis Reveals Potential Modes of Orthogonal Drug Synergy

**Figure 4:**
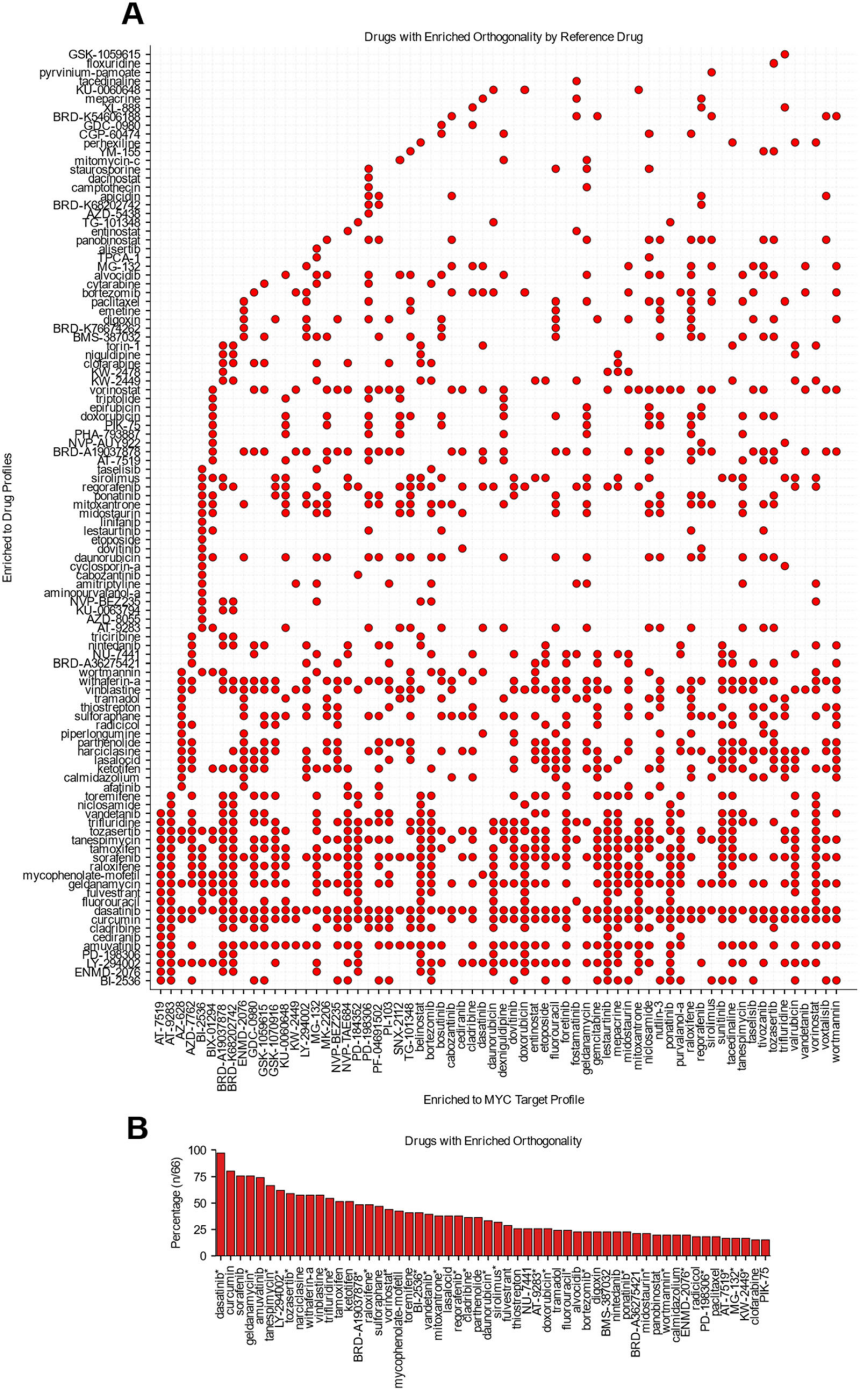
Landscape of predicted anti‐MYC synergistic drug combinations. (A) Binary heatmap showing orthogonality score enrichment for compounds previously identified as significantly enriched in the target profile. (B) Compounds with enriched orthogonality scores across > 10 drugs previously identified as strongly enriched for the MYC Target Profile. (*) marks compounds also enriched in the 70 potential MYC antagonists.

We noticed that for all the 70 prioritized MYC antagonist candidates, none of them can fully copy the molecular phenotype of MYC LOF (Supporting Figure 4B). In fact, the highest concordance of single agents is less than 40%. Knowingly, MYC is a promiscuous transcription factor regulating approximately 15% of the transcriptome, making it challenging to suppress its full target activity utilizing only single‐agent therapies [30, 31, 32]. Therefore, we hypothesized that we could increase the therapeutic coverage of MYC's activity by utilizing combination therapies to target multiple independent oncogenic pathways simultaneously, which also has the benefit of potentially reduced dosing requirements and delaying drug resistance development [33]. To address the incomplete coverage of MYC LOF‐associated genes by single agents, we decided to use an orthogonality‐based in‐silico screening strategy to further identify compounds that can synergize with the 70 MYC antagonist candidates through complementary transcriptional responses (Figure 4A; Supporting Figure 4A)

Here, we implemented an orthogonality scoring metric, to identify compounds with high concordance to the MYC LOF target profile yet low concordance with a reference drug profile (Supporting Figure 4A, see Methods) [12]. We hypothesized that these identified compounds may have different mechanisms than the given drug profile which may increase the combination's total coverage of MYC target pathways. From the 70 compounds enriched in Figure 3E, we selected six with prior literary evidence of MYC antagonism: vorinostat, entinostat, LY‐294002, sirolimus, geldanamycin, and belinostat [25, 34, 35] (Supporting Figure 4A) as representative case studies. Although these compounds were significantly enriched in more than three cell lines, none achieved broad coverage across MYC‐regulated genes (Supporting Figure 4B). This suggests that combining them with orthogonally acting agents could expand gene coverage and strengthen MYC pathway suppression. Consistently, compounds with high orthogonality scores also displayed strong concordance to the MYC LOF target profile (Supporting Figure 4A), indicating that MYC antagonists can regulate distinct subsets of MYC LOF signature genes.

Among the 70 enriched compounds, dasatinib, a dual Abl/Src kinase inhibitor, emerged as the most broadly orthogonal, displaying potential synergy with 98% of the other enriched single agents (Figure 4A, B). In monotherapy, dasatinib decreased MYC levels in K562 and LAMA84 cells in a dose‐dependent manner at 24 h, and, when in combination with vorinostat, induces a greater cell cycle arrest response [36]. Furthermore, combination of dasatinib and bortezomib in multiple myeloma cells was also identified as synergistic and MYC was identified as the top downregulated gene [37]. Likewise, in SK‐BR‐3 (HER + ) breast cancer cells, which display high levels of MYC, dasatinib showed synergy with both sirolimus and LY‐2940002 [38].

LY‐294002 also demonstrated broad orthogonality, with synergy predicted against > 60% of the other enriched compounds (Figure 4B). Consistent with the findings of Stathias et al [12], this PI3K/BET dual inhibitor was enriched for orthogonal synergy with the aurora kinase inhibitor GSK‐1070916, reinforcing this combination as a candidate for MYC‐inhibition‐based strategies. Furthermore, as both LY‐294002 and sirolimus were enriched in Figure 3E and highly orthogonality with each other (Figure 4A), prior findings in leukemia cells show that their combination downregulated MYC and cyclin D1 proteins to further decrease cell cycle regulating protein levels [39].

Single‐agent HDAC inhibitors which were enriched in Figure 3E, including vorinostat, belinostat, entinostat, and racemic Trichostatin A (BRD‐A19037878), also showed strong synergy with other enriched single agents (Figure 4B), especially HSP90 inhibitors such as geldanamycin and tanespimycin (Figure 4A). Although their synergy has not been reported in a MYC‐specific context, drug synergy has been demonstrated in MDAMB‐231 cells [40]. Additionally, in K562 cells, combined treatment with vorinostat and geldanamycin suppresses STAT5 activity [41]. Since STAT5 binds strongly to the MYC super‐enhancer regions E3 and E4, this suppression may contribute to MYC depletion [42]. Therefore, these findings collectively suggest that this orthogonality analysis may capture clinically relevant synergies for MYC antagonism.

**Figure 5:**
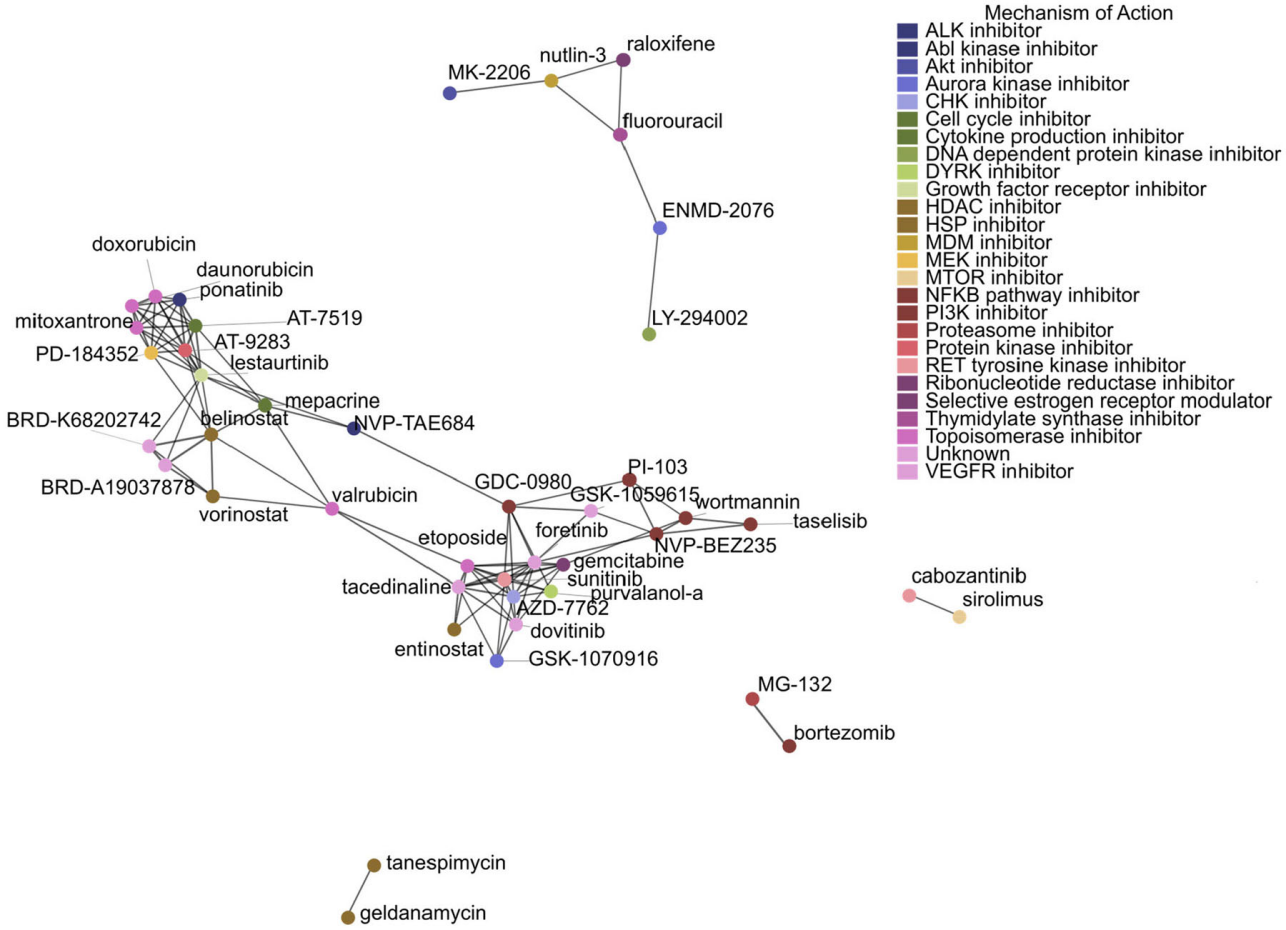
Functional drug network. Network constructed from the Jaccard similarity matrix, with nodes colored by mechanism of action according to CMAP annotations.

Several unannotated compounds also showed high concordance with the MYC LOF profile, prompting us to infer their functional capabilities as well as infer novel functions of established compounds. We hypothesized that drugs of similar functions should have similar synergistic drug partners. To measure the similarity of these synergistic drug combinations for all 70 single agents enriched in Figure 3E, we calculated the Jaccard similarity of their orthogonality enrichment profiles (Figure 4A; Supporting Figure 5A). We then constructed a network of similarity profiles (Figure 5) where compounds with similar synergistic drug combinations should be more closely related. We observed that many compounds with shared mechanisms of action tended to cluster together indicating that clusters may have a functional similarity (Supporting Figure 5D).

By further applying network analysis, we revealed six distinct clusters, each potentially corresponding to a unique functional aspect among these compounds (Supporting Figure 5B). Cluster 1 is primarily the tyrosine kinase inhibitor family and Cluster 2 is shared between HDAC and topoisomerase inhibitors (Supporting Figure 5D). The unannotated compound in Cluster 1, GSK‐1059615, is identified as a PI3K and MTOR dual inhibitor which, in gastric cancer cells, potently inhibited cell growth, survival, proliferation, and the cell cycle potentially decoupling MYC from its control over cell proliferation [43].

To identify compounds with potential dual or bridging functions, we then calculated the betweenness and degree for each drug node (Supporting Figure 5C). Valrubicin, GDC‐0980, lestaurtinib, and NVP‐TAE684 all exhibited high betweenness between Clusters 1 and 2 with NVP‐TAE684 and GDC‐0980 linking PI3K inhibitors to topoisomerase inhibitors, valrubicin and lestaurtinib linking HDAC inhibitors to topoisomerase inhibitors (Figure 5, Supporting Figure 5C). For NVP‐TAE684, this anaplastic lymphoma kinase (ALK) inhibitor, a subfamily of tyrosine kinase inhibitors, is able to induce G2/M phase cell cycle arrest and apoptotic cell death across multiple pancreatic cancer cell lines, particularly the fast‐growing COLO‐357, which is also a known function of topoisomerase II inhibitors and blocks MYC's promotions of the cell cycle [44, 45]. In one study, 100 nM of lestaurtinib impaired cell proliferation and caused an accumulation of DNA double strand breaks, blocked cell cycle progression, and activated the TP53 superfamily in vitro and in vivo, both of which may be similar to functions of topoisomerase and HDAC inhibitors [46]. Furthermore, mepacrine (quinacrine) is traditionally an anti‐malarial drug yet was identified in this screen as a bridging compound between HDAC and kinase inhibitor clusters, raising the possibility of it acting as a multitargeted therapeutic. A study previously demonstrated that mepacrine induced G0/G1 cell cycle arrest and apoptosis in diffuse large B‐cell lymphoma cell lines in a dose‐dependent manner and downregulate MYC cyclin‐dependent kinase 4/6 expression [47]. These bridging compounds warrant further experimental validation to evaluate their potential as multifunctional MYC antagonists.

## 4 Discussion

In this study, by utilizing a MYC loss‐of‐function mutant, we generated a transcriptional target profile to screen for compounds that may antagonize MYC activity. Using a normalized concordance metric, we evaluated drug‐induced transcriptional profiles from 10 CMAP cell lines for similarity to the MYC target profile and applied an enrichment analysis to assess whether compounds were significantly enriched at the top of the concordance rankings. To increase the robustness and biological interpretability of this analysis, both the full MYC target profile and pathway‐level approaches were employed in tandem.

Among the compounds significantly enriched in both methods, 70 were identified as recurrent hits in three or more cell lines. An orthogonality analysis was then applied to these 70 compounds to explore potential for drug synergy, with the rationale that compounds acting through independent mechanisms would have nonoverlapping gene expression effects. Furthermore, functional clustering of enrichment results enabled the generation of hypotheses regarding the mechanisms of action for uncharacterized compounds and highlighted possible new mechanisms for known agents in the context of MYC inhibition.

Within these 70 consistently enriched compounds, multiple known MYC antagonists were identified, including vorinostat, geldanamycin, sirolimus, and belinostat [25, 26] This recovery of indirect MYC inhibitors supports the validity of the concordance‐based screening approach. Notably, some well‐characterized MYC inhibitors such as JQ1 were not enriched, likely due to insufficient transcriptional activation as reflected by a lack of drug‐induced samples with a TAS ≥ 0.4. This underscores a key limitation in CMAP where high‐quality transcriptional signatures are not uniformly available for all compounds, especially those that exert subtle or delayed effects.

Although some topoisomerase inhibitors, including doxorubicin and daunorubicin, were enriched, it remains unclear whether this reflects MYC‐specific antagonism or nonspecific cytotoxicity via DNA damage. Interestingly, the absence of enrichment for other genotoxic agents such as paclitaxel, or cell‐cycle regulators like palbociclib, suggests some degree of specificity in the MYC‐associated transcriptional responses (Figure 3E).

Collectively, these findings demonstrate an in‐silico strategy capable of identifying MYC‐antagonistic compounds and generating a predictive network of potential synergistic drug combinations. This computational pipeline may be broadly applicable to other oncogenic profiles beyond MYC. However, several limitations must be acknowledged. First, the CMAP data set is unevenly sampled across cell lines and compounds limiting statistical power for generalization and internal replication. Second, the MYC LOF target profile used in this study was derived from U2OS cells, an osteosarcoma line with unique transcriptional and epigenetic characteristics. Generalizing these results to other tumor types requires validation in additional MYC‐dependent models.

Future efforts should aim to validate top novel candidate compounds and combinations in vitro, particularly in MYC‐addicted cancer cell lines. Additionally, this framework can be adapted to assess other oncogenic drivers (e.g., KRAS, NRF2, STAT3) or used in patient‐derived datasets for precision oncology applications. The use of orthogonality and normalized concordance scoring together offers a scalable, rational approach to prioritizing drug combinations, which can supplement or replace more labor‐intensive brute‐force drug combination screens.

## Author Contributions

Anthony Aceto and Yue Wang conceived and designed the study. Anthony Aceto performed the experiments. Anthony Aceto and Yue Wang drafted the manuscript. All authors reviewed and approved the final manuscript.

## Conflicts of Interest

The authors declare no conflicts of interest.

## Supporting information


**Supporting Figure 1 –** Number of samples per cell line, showing CMAP signature replicates and unique compounds tested at 10 µM for 24 h. **Supporting Figure 2 – (A)** Concordant drugs were defined as those with a concordance score > 0 and false discovery rate (FDR) ≤ 0.05. The concordance rate (CR) was calculated as the proportion of significant, concordant drugs relative to the total number of compounds in each cell line. **(B)** An independent t‐test comparing concordant drugs from upregulated and downregulated pathways across all cell lines yielded a t‐statistic of 3.93 (*p* = 1.43 × 10⁻⁴, α ≤ 0.05). **(C)** Gene‐level CRISPR knockout survival probabilities were obtained from the DepMap database and averaged across cancer cell lines, excluding HEPG2, which lacked CRISPR data. Distributions are shown for each hallmark pathway, with pathways colored by the GSEA NES value of the target profile**. Supporting Figure 3 – (A)** Effect of varying the enrichment threshold (number of cell lines in which a drug is enriched) on the confidence of MYC activity labels. Cell lines were classified as high or low MYC activity based on median MYC Targets v1 expression in the CCLE, and the proportion of significant drugs relative to all GDSC‐compatible compounds was calculated for each label. **(B)** Impact of varying the proportion of cell lines classified as high or low MYC activity for drugs enriched in ≥ 6 cell lines. **Supporting Figure 4 – (A)** Scatterplots showing concordance values for six drug‐induced expression profiles relative to the MYC Target Profile. **(B)** Concordance of aggregate drug profiles with the MYC Target Profile. **Supporting Figure 5 – (A)** Pairwise comparison of enrichment profiles using the Jaccard similarity index. **(B)** Network clusters identified with the greedy modularity communities algorithm. **(C)** Centrality metrics, including betweenness and degree, for all nodes in each cluster. **(D)** Mechanism‐of‐action (MoA) abundances within the identified clusters.

## Data Availability

This study analyzes previously published RNAseq data available in the GEO repository under accession number GSE273777. This study does not report the original code. Any additional information required to reanalyze the data reported in this study is available from the lead contact upon request.
