# Supplementary material for: An Integrative Drug‐Induced Transcriptomic Analysis Identifies Novel MYC Antagonists and Potential Synergistic Drug Combinations
Source: Mol Carcinog. 2025 Sep 30;64(12):2052–62. doi: 10.1002/mc.70044 (PMC12639535; doi:10.1002/mc.70044)
Supplement: Supplementary file 1 — Supporting Figure 1 – Number of samples per cell line, showing CMAP signature replicates and unique compounds tested at 10 µM for 24 h. Supporting Figure 2 – (A) Concordant drugs were defined as those with a concordance score > 0 and false discovery rate (FDR) ≤ 0.05. The concordance rate (CR) was calculated as the proportion of significant, concordant drugs relative to the total number of compounds in each cell line. (B) An independent t‐test comparing concordant drugs from upregulated and downregulated pathways across all cell lines yielded a t‐statistic of 3.93 (p = 1.43 × 10⁻⁴, α ≤ 0.05). (C) Gene‐level CRISPR knockout survival probabilities were obtained from the DepMap database and averaged across cancer cell lines, excluding HEPG2, which lacked CRISPR data. Distributions are shown for each hallmark pathway, with pathways colored by the GSEA NES value of the target profile. Supporting Figure 3 – (A) Effect of varying the enrichment threshold (number of cell lines in which a drug is enriched) on the confidence of MYC activity labels. Cell lines were classified as high or low MYC activity based on median MYC Targets v1 expression in the CCLE, and the proportion of significant drugs relative to all GDSC‐compatible compounds was calculated for each label. (B) Impact of varying the proportion of cell lines classified as high or low MYC activity for drugs enriched in ≥ 6 cell lines. Supporting Figure 4 – (A) Scatterplots showing concordance values for six drug‐induced expression profiles relative to the MYC Target Profile. (B) Concordance of aggregate drug profiles with the MYC Target Profile. Supporting Figure 5 – (A) Pairwise comparison of enrichment profiles using the Jaccard similarity index. (B) Network clusters identified with the greedy modularity communities algorithm. (C) Centrality metrics, including betweenness and degree, for all nodes in each cluster. (D) Mechanism‐of‐action (MoA) abundances within the identified clusters. [file MC-64-2052-s001.docx]

**Supplementary**

**S1**


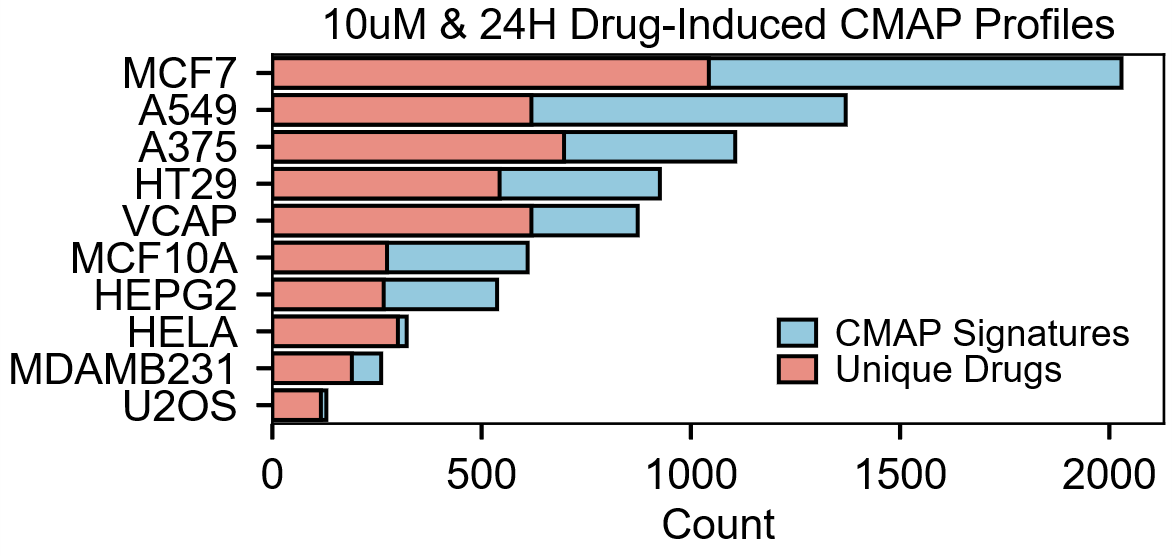


**Supplementary Figure 1 –** Number of samples per cell line, showing CMAP signature replicates and unique compounds tested at 10 µM for 24 h.

**S2**


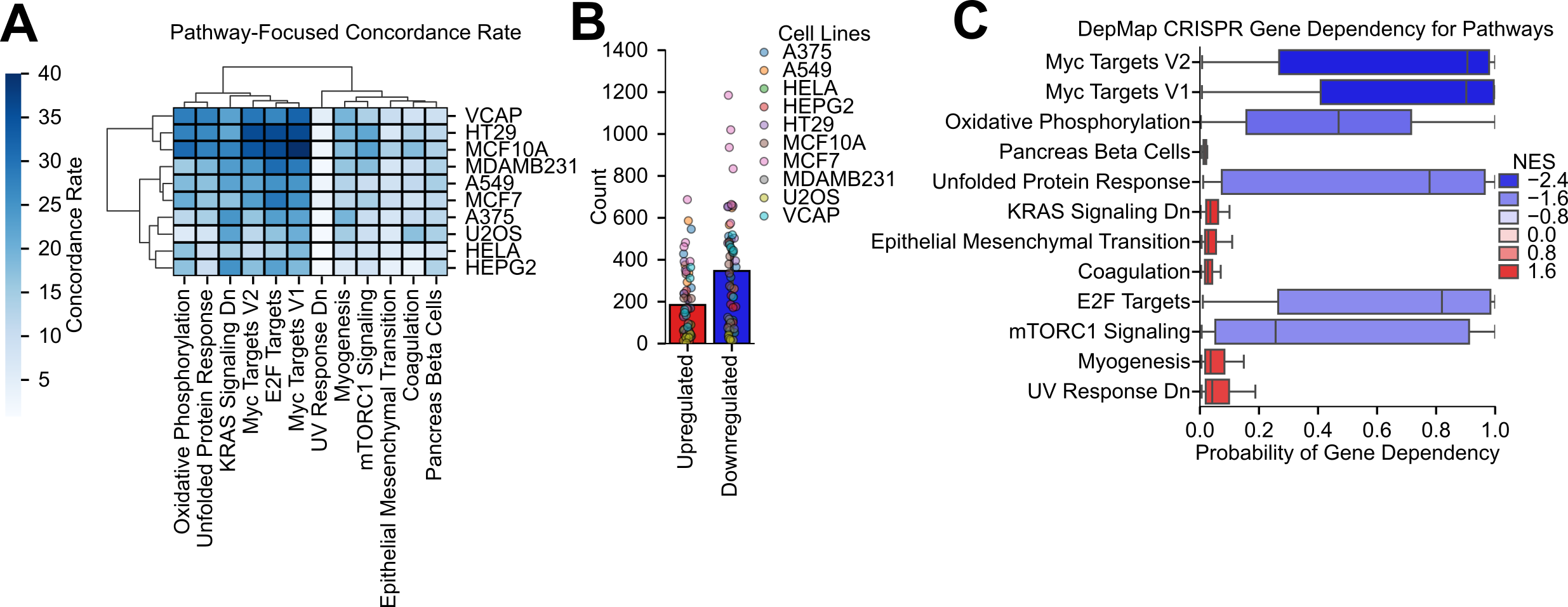


**Supplementary Figure 2 – (A)** Concordant drugs were defined as those with a concordance score > 0 and false discovery rate (FDR) ≤ 0.05. The concordance rate (CR) was calculated as the proportion of significant, concordant drugs relative to the total number of compounds in each cell line. **(B)** An independent t-test comparing concordant drugs from upregulated and downregulated pathways across all cell lines yielded a t-statistic of 3.93 (p = 1.43 × 10⁻⁴, α ≤ 0.05). **(C)** Gene-level CRISPR knockout survival probabilities were obtained from the DepMap database and averaged across cancer cell lines, excluding HEPG2, which lacked CRISPR data. Distributions are shown for each hallmark pathway, with pathways colored by the GSEA NES value of the target profile**.**

**S3**

**
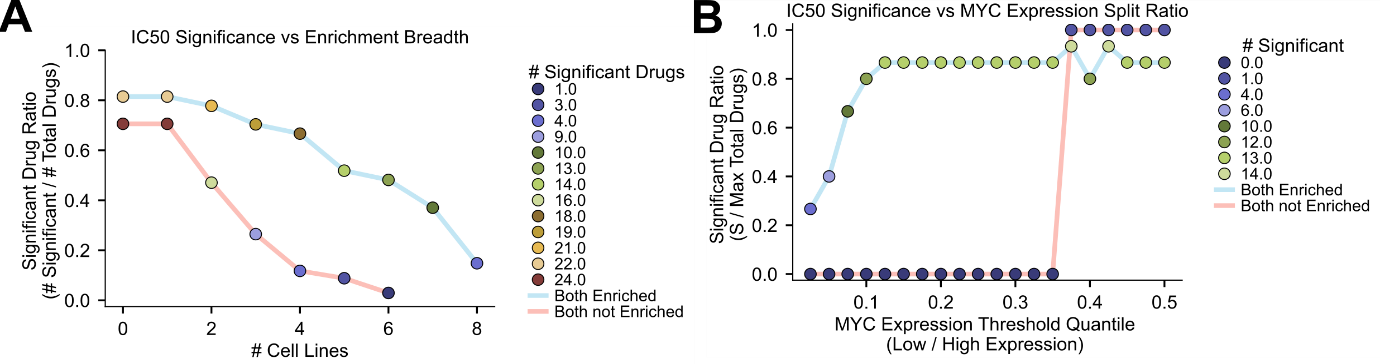
**

**Supplementary Figure 3 – (A)** Effect of varying the enrichment threshold (number of cell lines in which a drug is enriched) on the confidence of MYC activity labels. Cell lines were classified as high or low MYC activity based on median MYC Targets v1 expression in the CCLE, and the proportion of significant drugs relative to all GDSC-compatible compounds was calculated for each label. **(B)** Impact of varying the proportion of cell lines classified as high or low MYC activity for drugs enriched in ≥ 6 cell lines.

**S4**


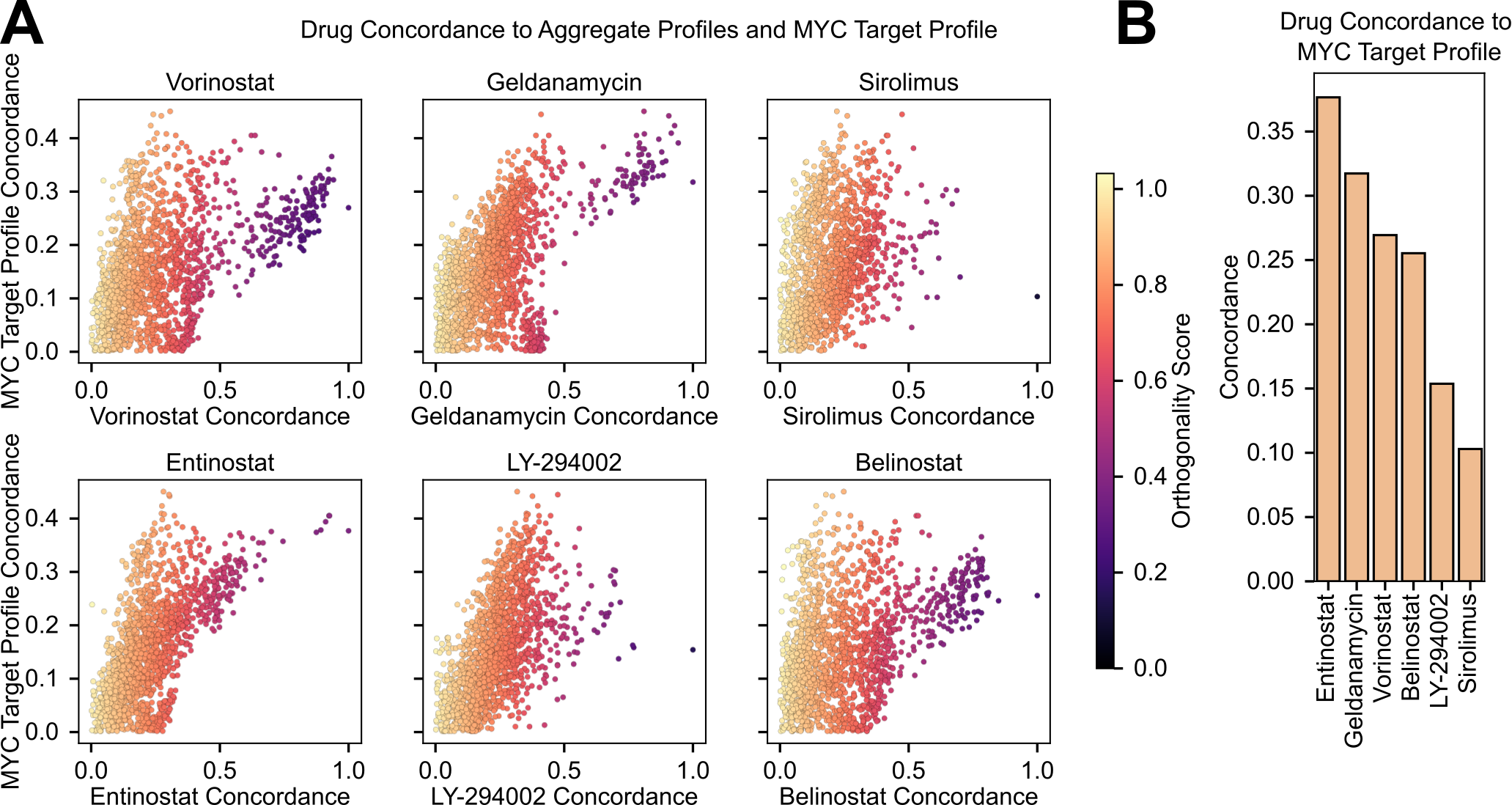


**Supplementary Figure 4 – (A)** Scatterplots showing concordance values for six drug-induced expression profiles relative to the MYC Target Profile. **(B)** Concordance of aggregate drug profiles with the MYC Target Profile.

**S5**

**
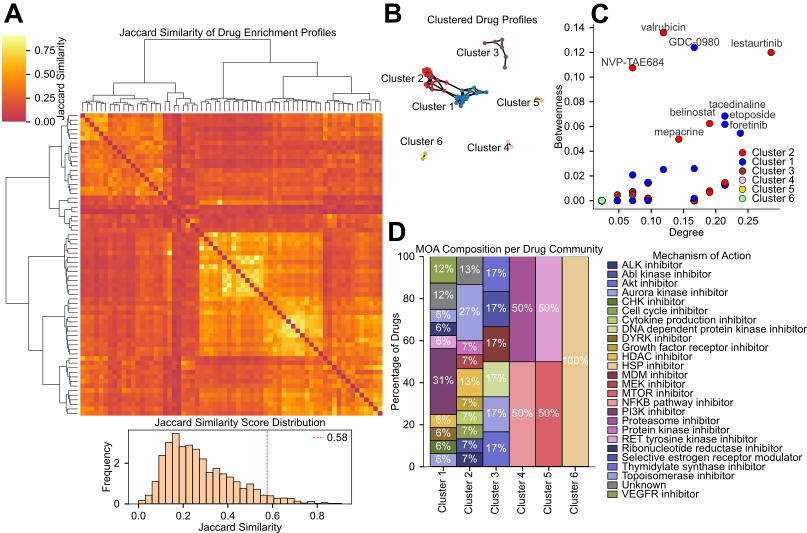
**

**Supplementary Figure 5 – (A)** Pairwise comparison of enrichment profiles using the Jaccard similarity index. **(B)** Network clusters identified with the greedy modularity communities algorithm. **(C)** Centrality metrics, including betweenness and degree, for all nodes in each cluster. **(D)** Mechanism-of-action (MoA) abundances within the identified clusters.
